# Moral Medicine

**Published:** 1877-08

**Authors:** 


### HALL'S JOURNAL OF HEALTH.

Our Legitimate Scope is almost boundless: for whatever begets pleasurable and harmless feelings, promotes Health; and whatever induces disagreeable sensations, engenders Disease.

WE AIM TO SHOW HOW DISEASE MAY BE AVOIDED, AND THAT IT IS BEST, WHEN SICKNESS COMES, TO TAKE NO MEDICINE WITHOUT CONSULTING A PHYSICIAN.

Vol. 24.] AUGUST, 1877. [No. 8.

### MORAL MEDICINE.

DRUGGERY is not the only physic. There are means of curing disease and averting sickness, quite as efficient as any pill or potion ever sold over the apothecary's counter. The medicine of the mind is as powerful for life and death, as the flash of the lightning, or the bullet of the carbine. The thunder-bolt kills instantly; so may a mental emotion. There are slow poisons which eat out the life, piece-meal, in the agonies of years; and many an unrevealed sorrow has there been, to waste away its victim in the tedious progress of weary weeks and months of grief. There is no stimulant more full of health than a hearty laugh. There is not a tonic in all creation which gives such perennial vigor as that of a conscience void of offence toward God and man. Better than any balm of ancient Gilead are the reflections of a well-spent life; of a conscious integrity of purpose pervading every business transaction from early joyous youth to a genial old age.

Let the reader, then, turn again to the pages of the *Journal of Health* [illegible] experiment for himself as to the virtue there is in "Living t[illegible] Purpose." Let him feel that *he* "is wanted" to do somewhat [illegible]ward raising humanity from its low estate to greater hig[illegible], and that without his aid, the grand work will be proporti[illegible]ally retarded. Let him be admonished in his progress down the river of time, lest he be unconsciously "drifting" upon sunken shoals or more treacherous quick-sands. Let him feel that there is no moth known on earth, which so effectually eats out all that is noble, and generous, and manly in the heart, as the "greed of gold." Thus let his eye run from article to article, and see if in the practice of them there is not an enduring virtue beyond that of the pestle and the spatula; more subtle and life-inspiring than Chemistry ever claimed.

